# Traversing the Cell Wall: The Chitinolytic Activity of *Histoplasma capsulatum* Extracellular Vesicles Facilitates Their Release

**DOI:** 10.3390/jof9111052

**Published:** 2023-10-27

**Authors:** Alessandro F. Valdez, Taiane Nascimento de Souza, Jhon Jhamilton Artunduaga Bonilla, Daniel Zamith-Miranda, Alicia Corbellini Piffer, Glauber R. S. Araujo, Allan J. Guimarães, Susana Frases, Alana Kelyene Pereira, Taicia Pacheco Fill, Igor L. Estevao, Angel Torres, Igor C. Almeida, Joshua D. Nosanchuk, Leonardo Nimrichter

**Affiliations:** 1 Departamento de Microbiologia Geral, Instituto de Microbiologia Paulo de Góes, Universidade Federal do Rio de Janeiro, Rio de Janeiro 21941-902, RJ, Brazil; alessandro.valdez@micro.ufrj.br (A.F.V.); taiane.desouza@einsteinmed.edu (T.N.d.S.); jhab61@hotmail.com (J.J.A.B.); aliciacpiffer@gmail.com (A.C.P.); 2 Department of Microbiology and Immunology, Albert Einstein College of Medicine, Bronx, NY 10461, USA; daniel.zamithmiranda@einsteinmed.edu; 3 Division of Infectious Diseases, Department of Medicine, Albert Einstein College of Medicine, Bronx, NY 10461, USA; 4 Unité Biologie des ARN des Pathogènes Fongiques, Départament de Mycologie, Institut Pasteur, Université Paris Cité, F-75015 Paris, France; 5 Laboratório de Biofísica de Fungos, Instituto de Biofísica Carlos Chagas Filho, Universidade Federal do Rio de Janeiro, Rio de Janeiro 21941-902, RJ, Brazil; glauber@biof.ufrj.br (G.R.S.A.); susanafrases@biof.ufrj.br (S.F.); 6 Instituto Biomédico, Departamento de Microbiologia e Parasitologia—MIP, Universidade Federal Fluminense, Niterói 24210-130, RJ, Brazil; allanguimaraes@id.uff.br; 7 Rede Micologia, RJ, FAPERJ, Rio de Janeiro 21941-902, RJ, Brazil; 8 Instituto de Química, Universidade Estadual de Campinas, Campinas, São Paulo 13083-970, SP, Brazil; alanakelyene@gmail.com (A.K.P.); taicia@gmail.com (T.P.F.); 9 Department of Biological Sciences, Border Biomedical Research Center, University of Texas El Paso, El Paso, TX 79902, USA; ilestevaoda@utep.edu (I.L.E.); atorres118@utep.edu (A.T.); icalmeida@utep.edu (I.C.A.)

**Keywords:** *Histoplasma capsulatum*, histoplasmosis, chitinase, caffeine, methylxanthine, extracellular vesicles, exosomes

## Abstract

*Histoplasma capsulatum* is the causative agent of histoplasmosis. Treating this fungal infection conventionally has significant limitations, prompting the search for alternative therapies. In this context, fungal extracellular vesicles (EVs) hold relevant potential as both therapeutic agents and targets for the treatment of fungal infections. To explore this further, we conducted a study using pharmacological inhibitors of chitinase (methylxanthines) to investigate their potential to reduce EV release and its subsequent impact on fungal virulence in an in vivo invertebrate model. Our findings revealed that a subinhibitory concentration of the methylxanthine, caffeine, effectively reduces EV release, leading to a modulation of *H. capsulatum* virulence. To the best of our knowledge, this is the first reported instance of a pharmacological inhibitor that reduces fungal EV release without any observed fungicidal effects.

## 1. Introduction

*Histoplasma capsulatum* (Hc) is a thermally dimorphic fungus distributed worldwide, causing histoplasmosis in both immunocompetent and immunocompromised individuals. The fungus thus meets the criteria of a primary pathogen [1]. The prognosis of histoplasmosis hinges on factors such as the inoculum size, virulence of the infecting strain, and immune status of the infected individual [2]. This makes the situation particularly complex for immunocompromised individuals, who face a higher risk of disseminated and chronic pulmonary histoplasmosis, with mortality rates reaching up to 53% [1,2,3,4]. 

Antifungal therapies for histoplasmosis require prolonged administration, are costly, and their effectiveness has diminished due to emerging resistance in some strains isolated from patients [5,6]. This scenario is not unique to histoplasmosis. Many antifungal therapies face challenges due to pharmacological limitations and rising resistance. This has spurred increasing interest in alternative approaches, including drug repurposing and the development of nano- and micro-delivery drug systems [7]. Collectively, these challenges underscore the imperative to explore new treatment and prevention options for histoplasmosis and other fungal infections.

In this context, fungal extracellular vesicles (EVs) are a druggable target. EVs are described as a non-conventional secretion mechanism fungi utilize to release various biomolecules into the extracellular environment, including proteins, lipids, glycans, polysaccharides, nucleic acids, metabolites and pigments [8,9,10,11,12,13,14,15,16,17,18,19]. Using *C. albicans* EVs as an example, their diverse biological effects have been documented, from modulating biofilm production to providing immunization via EVs in insects and mice [9,15,17]. In *H. capsulatum*, studies have indicated that vesicular compounds are immunoreactive to sera from infected patients [20]. Additionally, the EV cargo in *H. capsulatum* yeasts can be modulated when treated with monoclonal antibodies (mAbs) targeting the heat shock protein 60 (Hsp60) [21]. Recent findings also demonstrate that *H. capsulatum* EVs influence interactions between yeasts and cells of the innate immune system, leading to reduced yeast killing rates by both macrophages and dendritic cells in vitro [22]. Moreover, the content of *H. capsulatum* EVs comprises mRNA transcripts that do not directly correlate with the cell’s most highly expressed transcripts. This suggests a regulated mechanism guiding these RNAs to the EVs [18]. Furthermore, fungal EVs have demonstrated the ability to adjust virulence in vivo [23,24,25,26]. Consequently, EVs, as well as the processes involved in their formation and release, emerge as promising targets for innovative therapeutic approaches. This underscores the importance of continued research in this area to fully exploit their potential.

Over the 16 years since fungal EVs were first described, [13] significant advances have been made in understanding their biology. However, a pivotal question remains: *how do these compartments traverse the intricate structure of the fungal cell wall?* Three primary, non-mutually exclusive hypotheses have been proposed to explain this phenomenon: (i) the influence of turgor pressure in the periplasmatic space; (ii) the existence of channels within the cell wall; and (iii) enzymatic activities contained in the EV cargo. The third hypothesis draws significant support from the proteomic analyses of molecules transported by EVs. Notably, among these are enzymes related to the hydrolysis of major cell wall polysaccharides, such as glucanases, mannosidases, and chitinases [16,20,21]. Chitinases, in particular, have been identified in EVs produced by *H. capsulatum*, and even though the functional diversity of fungal chitinases is not yet well understood, they are known to be crucially involved in the cell wall remodeling process and are essential for fungal growth and survival [27,28]. In fact, Goughenour et al. recently investigated the expression of eight chitinase encoding genes and characterized seven different active chitinases from the *H. capsulatum* proteome, which are hypothesized to be involved in a plethora of processes in both yeast and mycelial morphology [29].

In this study, we aim to confirm the presence of active chitinases in *H. capsulatum* EVs and explore the role of these enzymes in EV release using methylxanthines as pharmacological inhibitors. Methylxanthines are a class of molecules recognized as competitive inhibitors of fungal chitinases. They also exhibit antifungal activity at elevated concentrations [30,31]. We demonstrate that the deployment of methylxanthines can shed light on the EV release process through the cell wall and can be crucial for understanding EV involvement during infection. To our knowledge, no other pharmacological inhibitors associated with EV release have been identified, making it challenging to determine their genuine in vivo biological function. Although turbinmicin is believed to interfere with fungal vesicle-mediated trafficking [32], this effect is only observed at cytotoxic concentrations, as reported by the authors, constraining its utility for in-depth EV biology studies.

## 2. Materials and Methods

### 2.1. Fungal Strains 

The reference strain *H. capsulatum* G217B (ATCC 26032) was grown for 2 days in an orbital shaker (200 RPM) at 37 °C in Ham’s F-12 medium (Gibco™ #21700075, Billings, MT, USA) supplemented with glucose (18.2 g/L—Sigma-Aldrich G8270, St. Louis, MO, USA), glutamic acid (1 g/L—ISOFAR 509), HEPES (6 g/L—Gibco 11344-041) and cysteine (8.4 mg/L—Pierce 44889), as described previously [33]. *C. albicans* 90028 (ATCC 90028), *C. parapsilosis* GA1 (clinical isolate) and *S. cerevisiae* 288C (ATCC 204508) were grown in Sabouraud broth (Difco 238230) in the same conditions. Treatment with the methylxanthines (caffeine, C0750; pentoxifylline, P1784; aminophylline, A1755—Sigma-Aldrich) was accomplished by diluting a 20 mM stock solution prepared in the correspondent media to the desired final concentrations.

### 2.2. Fluorescence Microscopy

*H. capsulatum*, *C. albicans*, *C. parapsilosis* and *S. cerevisiae* yeasts were harvested and washed with phosphate-buffered saline (PBS—137 mM NaCl, 2.7 mM KCl, 8 mM Na_2_HPO_4_ and 2 mM KH_2_PO_4—_pH 7.3 ± 0.1) upon cultivation. The yeasts were enumerated and 5 × 10^6^ cells were fixed in a solution of PBS/paraformaldehyde 4% for 30 min. The cells were then washed three times with PBS and co-incubated with WGA–rhodamine 10 µg/mL (30 min) and Uvitex 2B 5 µg/mL (10 min) at 37 °C. Yeasts were further washed with PBS after incubation and the samples were examined in an Axio Observer Z1 (Carl Zeiss, Oberkochen, Germany) using a 600× magnification. Images were analyzed via Zen Blue (Carl Zeiss) and Adobe Photoshop CS6 (Adobe Systems Software–Version 13.0.1). 

### 2.3. EV Isolation

EVs were isolated from the *H. capsulatum* growth supernatant, as described previously (Rodrigues et al., 2007) [13]. In brief, *H. capsulatum* supernatants from 48 h cultures were subjected to sequential centrifugation steps (4000× *g* and 15,000× *g*, 15 min, at 4 °C). The remaining yeasts and debris were removed by an additional step of supernatant filtration using a 0.45 μm membrane filter (Merck Millipore, Burlington, MA, USA). Cell-free supernatants were then concentrated approximately 20-fold using an Amicon ultrafiltration system (cut-off, 100 kDa, Millipore) and then centrifuged at 100,000× *g* for 1 h at 4 °C. Supernatants were then discarded and EV-containing pellets washed twice with 0.1 M PBS pH 7.4 at 100,000× *g* for 1 h at 4 °C. EVs from the cells treated with caffeine were isolated using the same procedure. EV quantification was performed either by total protein dosage with the Pierce^TM^ (Waltham, MA, USA) BCA Protein Assay kit (Pierce 23227) or nanoparticle tracking analyses (described in Section 2.8). 

### 2.4. Chitinase Activity

Chitinase enzymatic activity was determined via a fluorometric assay based on an adaptation of a protocol previously described by Zhu et al., 2004 [34]. Using a fluorogenic chitinase substrate, 4-methylumbelliferyl-β-D-N,N’,N’-triacetylchitotriose (4MU-(GlcNAc)_3_), which upon reaction releases a fluorescent product, 4-methylbelliferone. For biochemical reactions, 50 µL of culture supernatant or EV samples (50 µg of protein) were added to 25 µL of substrate (50 µM) and 25 µL McIlvaine’s buffer (pH 6.0), and the reaction mixture was then incubated at 37 °C for 30 min. The endpoint fluorescence was measured using a plate reader (SpectraMax i3, Molecular Devices, California, CA, USA—365 nm excitation and 460 nm emission), and the results were expressed in percentual median fluorescence activity (% MFI). Inhibition of chitinase activity in *H. capsulatum* EVs was achieved by incubating EVs (50 µg of protein) with 5 mM of each methylxanthine for 1 h at 37 °C before performing the fluorometric assay. *Streptomyces griseus* purified chitinase (0.5 × 10^−4^ U/mL—Sigma-Aldrich C6137) was used as a positive control. Activity assays were performed in triplicate. 

### 2.5. Fungal Growth Inhibition

Growth inhibition assays were performed using 200 μL of 5 × 10^5^ *H. capsulatum* yeast/mL in Ham’s F-12, incubated with different concentrations of caffeine (1, 5 and 10 mM) or in the absence of this drug for control growth curves. Plates were incubated at 37 °C for approximately 4 days in a Bioscreen (LabSystems Oy, Vantaa, Finland) with continuous shaking, with 600 nm absorbance readings recorded every hour, and values plotted against time to obtain the growth curves for each individual condition. The area under the curve (AUC) was calculated as an integrative parameter of total growth, as described previously [35,36,37]. The values obtained in the presence of caffeine were individually compared to a control curve in its absence.

### 2.6. EV Transmission Electron Microscopy

Vesicle morphology and size were determined via transmission electron microscopy (TEM) using the negative staining technique [9]. In short, 5 μL EVs previously purified were adsorbed for 30 s to a Formvar film on a carbon-coated 300-mesh copper grid. Excess was removed using filter paper. Subsequently, the samples were stained with 2.5% uranyl acetate for 30 s. EVs were visualized using a transmission electron microscope (FEI Tecnai Spirit) operated at 120 kV. ImageJ software (version 1.53K) was used to measure the EV diameter. Data were analyzed with GraphPad Prism 7 software (GraphPad, La Jolla, CA, USA). 

### 2.7. Yeast Scanning Electron Microscopy 

The samples were washed three times in PBS and fixed in 2.5% glutaraldehyde (Electron Microscopy Sciences) in a sodium cacodylate buffer 0.1 M pH 7.2 ± 0.1 for 45 min at room temperature. Then, the cells were washed three times in a 0.1 M sodium cacodylate buffer pH 7.2 ± 0.1 containing 0.2 M sucrose and 2 mM MgCl_2_ (Merck Millipore), and adhered to glass coverslips (Paul Marienfeld GmbH & Co., Lauda-Königshofen, Germany) previously coated with 0.01% poly-L-lysine (Sigma) for 40 min. The adhered cells were then gradually dehydrated in an ethanol growing series 30, 50 and 70% for 5 min and 95% and 100% twice for 10 min (Merck Millipore). The coverslips were then critical-point-dried using an EM DPC 300 critical point drier (Leica, Wetzlar, Germany) and mounted on specimen stubs using a conductive carbon adhesive (Pelco Tabs™, Fresno, CA, USA). Next, the samples were coated with gold–palladium (10 nm) using the sputter method (Balzers Union, Balzers, Liechtenstein). Finally, the samples were visualized using a scanning electron microscope VEGA3 (Brno, Czech Republic) and images were collected with their respective software packages (version 4.2.30.0) (Essence™, Brooklyn, NY, USA).

### 2.8. ZetaView Nanoparticle Tracking Analysis

EV sizes and concentration were measured using the ZetaView nanoparticle tracking analyzer (NTA; Particle Metrix GmbH, Inning am Ammersee, Germany). For measurements, samples were diluted at 1:1000 in previously filtrated PBS (0.22 μm) for an optimal concentration range for the NTA software (ZetaView Software version 8.02.31, Particle Metrix GmbH). Software parameters were the temperature at 23 °C, the sensitivity of 30–85 frames per second (fps), a shutter speed of 55, and laser pulse duration equal to that of the shutter duration. Acquisition parameters were set to a minimum brightness of 20, a maximum size of 200 pixels, and a minimum size of 5 pixels. Polystyrene particles (Microtrac GmbH, Haan, Germany) with an average size of 100 nm were used to calibrate the instrument before the sample readings. Data were analyzed using the ZetaView software (version 8.02.31) and GraphPad Prism 7 (GraphPad, CA, USA). For size distribution graphs, the number of particles detected in each point was normalized to the total number of particles in each sample, and the results were expressed as the relative number of particles/mL. This normalization was performed to facilitate the visualization of the size distribution between the groups. The concentration of EVs per cell was calculated dividing the total number of particles detected in each isolate by the final number of cells in its respective 48 h culture. 

### 2.9. Virulence in Galleria Mellonella

To investigate the effect of caffeine treatment on fungal virulence and the direct impact of *H. capsulatum* EVs during histoplasmosis, three protocols were tested using *G. mellonella* larvae as a model. A total of 10 insects (0.3 g each) were used per experimental condition. Larvae were inoculated with 10 μL of fungal suspensions containing 5 × 10^8^ yeasts/mL (5 × 10^6^ yeasts/insect) into the hemocoel through the last proleg using a 30 G insulin syringe, as previously described [38]. First, we investigated whether the treatment of *H. capsulatum* yeasts with caffeine could impact virulence. *H. capsulatum* was cultivated in Ham’s F-12 medium supplemented or not with caffeine (1 mM) for 48 h, as described. Yeasts were washed with PBS, enumerated and immediately used to infect the larvae. PBS alone was used as a negative control. The mortality of the insects was accompanied for 15 days. Second, the yeasts of *H. capsulatum* were co-injected with EVs obtained from *H. capsulatum* cultivated in Ham’s-F12 (EV_CTL_—100 μg/mL), and the insect mortality followed for 15 days. Finally, to investigate whether EVs could reverse the virulence decrease observed for yeasts cultivated in the presence of caffeine, insects were co-injected with caffeine-treated yeasts (1 mM) and EV_CTL_ (100 μg/mL). Again, the death of insects was used as a read out. All larvae were placed in sterile Petri dishes and maintained in the dark at 37 °C. Larvae mortality was monitored daily per fifteen days. Death was assessed by the lack of movement in response to stimulation. Data were analyzed with GraphPad Prism 7 software (GraphPad, CA, USA) and the results represent the mean percentage survival of the larvae from all assays. Two independent experiments were made. 

### 2.10. EV Caffeine Content 

EVs from different samples of *H. capsulatum* and *C. albicans* were extracted following the protocol described by Costa et al., 2021 [19]. Briefly, the EVs were extracted using 0.5 mL HPLC-grade methanol and submitted to an ultrasonic bath for 1 h at room temperature. To detect caffeine in the vesicles, HPLC-MS/MS analyses were performed with a Waters Acquity UPLC system coupled to a Waters Micromass Quattro Micro TM API with electrospray ionization source and a triple–quadrupole analyzer. An SRM method was developed using an authentic analytical standard of caffeine and the optimal transition was established as *m/z* 195.1→41.6 and collision energy 25 eV. The analyses were performed in a positive ion mode, with a capillary voltage of 3.7 kV and cone voltage 24 V. Chromatographic separation was achieved using a Thermo Scientific (Waltham, MA, USA) column Accucore C18 2.6 µm (2.1 mm), and mobile phase B comprised 0.1% formic acid in acetonitrile (ACN). The method was optimized and conducted as follows: 98/2 up to 90/10 within 6 min, then up to 10/90 in 1 min, held for 5 min, and finally up to 98/2 within 1 min, and held for 2 min. The total run time was 10 min. The data were processed using Waters MassLynx v.41.

### 2.11. Proteomic Analysis of Fungal EVs

EVs from *H. capsulatum* yeasts treated or not with 1 mM caffeine were subjected to proteomic analysis via nano-high-performance liquid chromatography coupled to tandem mass spectrometry (nanoHPLC-MS/MS), essentially as previously described [39,40,41,42], with some modifications. Briefly, fungal EV samples (50 μg protein) were redissolved in 200 μL 100 mM Tris-HCl pH 7.0, containing 8 M urea (urea sample solution, USS). Protein digestion was performed with 200 μL of each sample and transferred to a 1.5 mL microtube containing 200 µL USS. Ten mM DL-dithiothreitol (Sigma-Aldrich) was added to reduce protein disulfide bonds and achieve complete protein unfolding. Following digestion with trypsin (Proteomics Grade, Cat. #T6567, Sigma-Aldrich, St. Louis, MO, USA) (enzyme/protein ratio at 1:50) using the FASP Protein Digestion Kit (catalog # ab270519, Abcam, Cambridge, MA, USA), the digested peptides were cleaned using a Phoenix clean-up kit (Cat. # P.O.00023, Preomics, Planegg, Germany), according to the manufacturer’s instructions. One microliter of trypsin-digested proteins, equivalent to 1 μg of protein, was loaded onto an Acclaim PepMap separation LC column (75 µm × 50 cm nanoViper, Cat. # 164942, Thermo Scientific) in a Dionex Ultimate 3000 RSLCnano (Thermo Fisher Scientific) with 96% solvent A (100% water, 0.1% formic acid) and 4% solvent B (100% acetonitrile, 0.1% formic acid), at a flow rate of 0.4 µL/min for 8 min. The flow rate was decreased to 0.3 µL/min at 10 min, to start the elution gradient. Solvent B was increased to 35% over 120 min. The gradient was increased to 95% solvent B over 15 min and maintained at a plateau for an additional 10 min. The gradient sharply decreased to 4% solvent B over 1 min for equilibration, with a flow rate at 0.4 µL/min, maintained for 24 min, thus ending the 180 min sample runtime. The eluted peptides were ionized with a Nanospray Flex Ion Source (Thermo Fisher Scientific) equipped with a 10 cm nanoESI emitter (20 μm ID, FOSSILIONTECH, Madrid, Spain). The column compartment was kept at 55 °C. One blank injection containing 4% ACN and 0.1% FA was performed between biological replicates to avoid the carryover of peptides from one sample to another, using a 60 min sawtooth gradient at 4–95% solvent B, and re-equilibrated at 4% solvent B for the next sample injection. All solvents used were of LCMS grade. 

Data were acquired in a positive mode using a Q Exactive Plus Hybrid Quadrupole-Orbitrap Mass Spectrometer (QE Plus, Thermo Fisher Scientific) set to data-dependent MS^2^. The mass spectrometer parameters were as follows: full MS resolution at 70,000; AGC target of 3 × 10^6^; and scan from 375 to 1500 *m*/*z* range. The top 10 data-dependent MS^2^ parameters were set to 17,500 resolution, maximum IT set to 60 ms, ACG target of 1 × 10^5^, NCE: 27, charged exclusion at unassigned, +1, +6–8, +>8 with the mass exclusion set to ON. The control samples (untreated) were run in biological duplicates and technical duplicates, whereas the caffeine-treated samples were run in biological triplicates and technical duplicates. 

Proteomic data analysis was initially performed using Proteome Discoverer (PD) v2.5.0.400 (Thermo Fisher Scientific), with an estimated false discovery rate (FDR) of 1%. Common contaminants such as trypsin autolysis fragments, human keratins and protein lab standards were included, along with in-house contaminants, which may be found in the cRAP contaminant database [43]. The *H. capsulatum* database (*Ajellomyces capsulatus*) was downloaded in the FASTA format on 24 March 2023, from UniProtKB (http://www.uniprot.org/ accessed 24 March 2023). The following parameters were used in the PD analysis: HCD MS/MS; fully tryptic peptides only; up to 2 missed cleavages; parent ion mass tolerance of 10 ppm (monoisotopic); and fragment mass tolerance of 0.6 Da (in Sequest) and 0.02 Da (monoisotopic). A filter of two high-confidence peptides per protein was applied for identifications. The PD dataset was further processed through Scaffold Q+ 5.2.2 (Proteome Software, Portland, OR, USA) to obtain the protein quantification and further analysis of the MS/MS spectra. A protein threshold of 95%, a peptide threshold of 90%, and a minimum of 2 unique peptides were used for protein validation.

## 3. Results

### 3.1. Histoplasma capsulatum Yeasts Displayed an Enhanced Exposure of Chitin Oligomers on the Cell Surface

**Figure 1:**
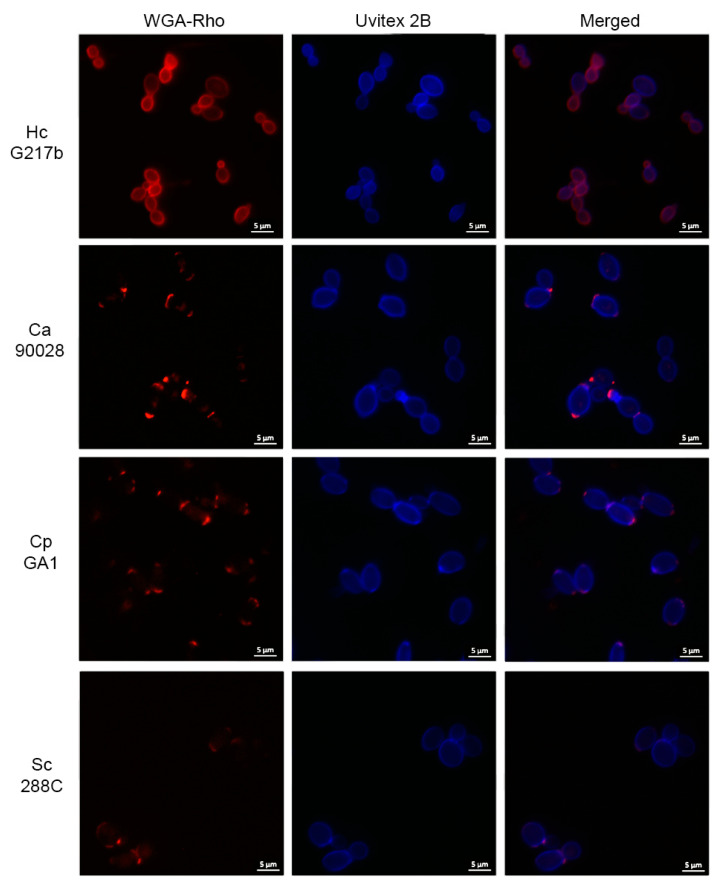
Chitin and chitin–oligomer distribution in the cell walls of *H. capsulatum*, *C. albicans*, *C. parapsilosis* and *S. cerevisiae*. Utilizing rhodamine-labeled WGA (red), the cell wall analysis indicates that *H. capsulatum* yeasts uniquely exhibit a homogenous ring pattern for chitin–oligomers, co-localizing with chitin, which is demarcated by Uvitex 2B (blue). Conversely, WGA binding appears in a distinct punctate arrangement at the budding neck regions of the cell walls for *C. albicans* (Ca), *C. parapsilosis* (Cp) and *S. cerevisiae* (Sc).

To identify and compare the distribution of chitin and chitin oligomers in *H. capsulatum*, *C. albicans*, *C. parapsilosis* and *S. cerevisiae*, we employed Uvitex 2B and wheat germ agglutinin–rhodamine (WGA–Rho), respectively. Uvitex 2B staining was similar in all fungal species, with a uniform ring pattern demarking the cell wall. However, WGA–Rho stained the yeasts in distinct patterns (Figure 1). *H. capsulatum* showed a homogeneous ring distribution for WGA labeling which co-localized with Uvitex 2B. In contrast, WGA labeling in *C. albicans*, *C. parapsilosis* and *S. cerevisiae* manifested in a punctate manner at the budding neck, as previously described in the literature [35]. The increased exposure of chitin oligomers in *H. capsulatum* suggests an augmented chitinase activity in this yeast compared to other fungal species.

### 3.2. Histoplasma capsulatum Showed a Higher Chitinase Activity in Its Supernatant and Extracellular Vesicles (EVs), Which Can Be Inhibited by Caffeine

**Figure 2:**
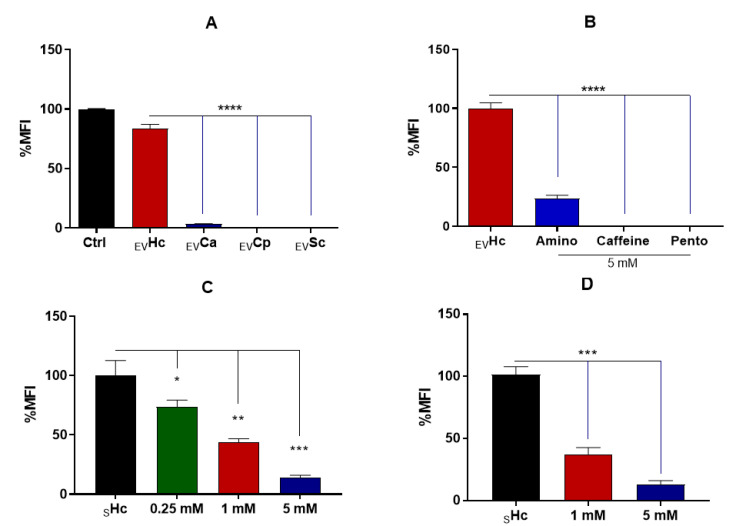
Chitinase activity on fungal EVs. Chitinase activity for *H. capsulatum*, *C. albicans*, *C. parapsilosis* and *S. cerevisiae* EVs. (**A**) Inhibition of chitinase activity in Hc EVs with the methylxanthines aminophylline, caffeine and pentoxifylline (5 mM). (**B**) Inhibition of chitinase activity in *H. capsulatum* supernatant (_S_Hc) by caffeine at 1 h (**C**) and 48 h (**D**), showing that the inhibitory effect was sustained. Statistical analysis was performed via a one-way ANOVA with Dunnett’s multiple-comparisons test (*, *p* < 0.05, **, *p* < 0.01, ***, *p* < 0.001, and ****, *p* < 0.0001).

We compared the chitinase activity of EVs released by the different fungal species. Under our experimental conditions, only the EVs from *H. capsulatum* and *C. albicans* demonstrated the capacity to hydrolyze the chitinase’s fluorogenic substrate (4MU-(GlcNAc)_3_), as shown in Figure 2A. Comparatively, the chitinase activity observed in *H. capsulatum* EVs was approximately 30-fold higher than that of EVs from *C. albicans*. Notably, the chitinase activity from fungal EVs was significantly reduced after treatment with three methylxanthines: aminophylline, caffeine and pentoxifylline (5 mM) (Figure 2B). Our results also revealed that a 1 h co-incubation of *H. capsulatum* yeasts with caffeine led to a dose-dependent decrease in the chitinase activity of the culture supernatant (Figure 2C). Yeasts were then co-incubated with different caffeine concentrations over 48 h and the chitinase inhibition evaluated. Although during the previous experiment, the concentration of 0.25 mM also significantly reduced chitinase activity, we opted for testing the concentrations where the activity was inhibited by at least 50%. Remarkably, caffeine concentrations of 1 and 5 mM were able to inhibit the enzyme for longer periods (Figure 2D). This indicates that these conditions could be employed to investigate the effect of caffeine on EV release.

### 3.3. Evaluation of Caffeine Antifungal Activity against H. capsulatum

**Figure 3:**
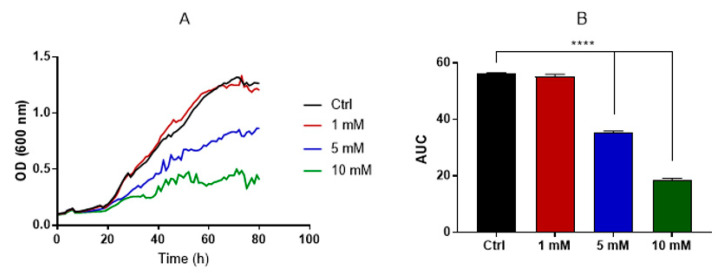
Evaluation of caffeine antifungal activity. Growth was monitored by OD using Bioscreen for 80 h (**A**). The area under the curve was calculated as an integrative parameter of the total growth and individual comparisons between each concentration, and the control group indicated that concentrations of 5 and 10 mM have antifungal activity (**B**). Statistical analysis was performed using a one-way ANOVA with Dunnett’s multiple-comparisons test (*p* < 0.05). ****, *p* < 0.0001.

**Figure 4:**
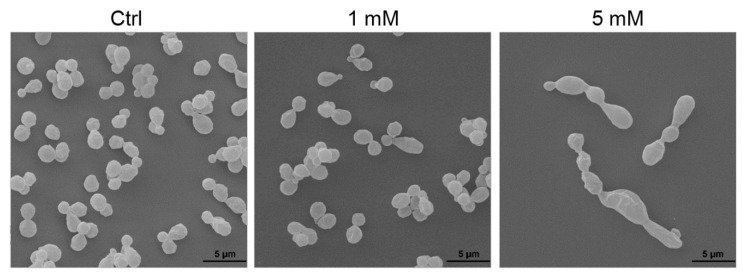
Scanning electron microscopy of *H. capsulatum* yeasts. The control and 1 mM caffeine-exposed yeasts did not show morphological differences. Yeasts exposed to 5 mM caffeine were substantially elongated and their budding was impaired.

Caffeine, along with other methylxanthine drugs, has demonstrated antifungal activity against *C. neoformans* and *A. fumigatus* [31]. By monitoring the optical density (OD) for approximately 3 days, we discerned the dose–effect relationship of caffeine on *H. capsulatum* growth. Using the area under the curve (AUC) as an integrative measure of total growth, individual comparisons between each caffeine concentration and the control group (without caffeine) revealed that the concentrations of 5 and 10 mM impeded *H. capsulatum* growth (Figure 3). Furthermore, scanning electron microscopy (SEM) images (Figure 4) showcased that yeasts treated with 5 mM caffeine were substantially elongated, with pronounced budding deficiencies. In contrast, there were no noticeable differences between the control and 1 mM caffeine groups.

### 3.4. Chitinase Inhibition Affects EV Release but Not Their Content

**Figure 5:**
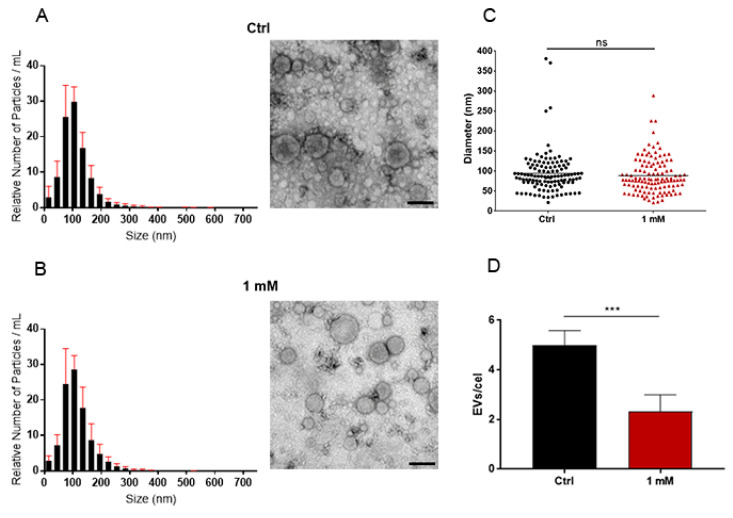
Influence of chitinase inhibition on EV release. Nanotracking analysis and negative staining transmission electron microscopy (TEM) of EVs released by the control (**A**) and yeast treated with 1 mM caffeine (**B**) did not show any differences in size distribution (**C**) or morphology. The number of EVs released by *H. capsulatum* yeast treated with caffeine was reduced by 50% (**D**). Scale bar = 100 nm. Statistical significance (*p* < 0.05) was assessed using an unpaired Student’s *t*-test; ns, non-significant; ***, *p* < 0.001.

**Figure 6:**
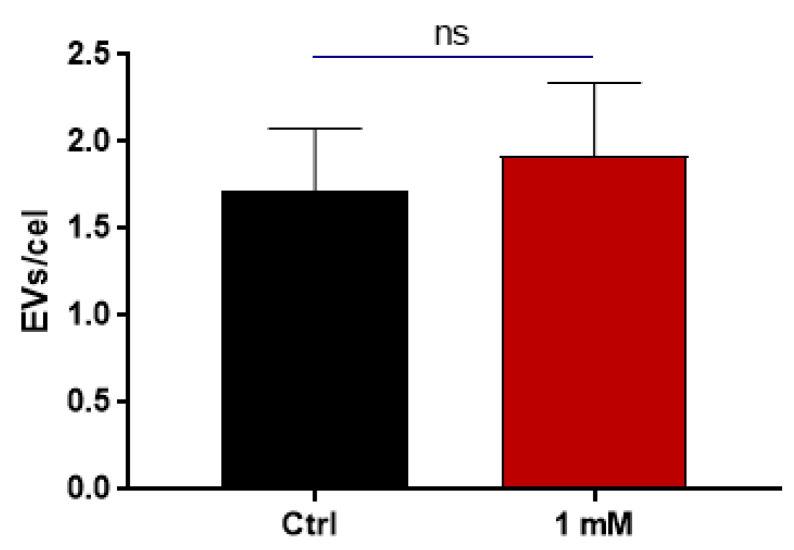
*C. albicans* extracellular vesicle quantification. EVs from *C. albicans* were isolated both in the presence and absence of 1 mM caffeine, and then quantified via NTA. The NTA data revealed no difference in the number of EVs released between the two conditions. Statistical significance (*p* < 0.05) was assessed using Student’s *t*-test; ns, non-significant.

Comparisons of isolated EVs were made between the control and 1 mM-treated caffeine groups. This was because the 1 mM caffeine treatment did not affect growth or cause major morphological changes. The physical attributes of the EVs were assessed using nanoparticle tracking analysis (NTA) and transmission electron microscopy (TEM) with negative staining, as described [9,44]. The observed size distribution was consistent with descriptions of fungal EVs in the literature. Notably, no significant size disparity was observed between the control and 1 mM caffeine-treated group. EV diameters from both groups averaged around 110 nm based on NTA and 90 nm via TEM (Figure 5). Remarkably, *H. capsulatum* yeasts treated with 1 mM caffeine released 50% fewer EVs per cell (Figure 5D). Furthermore, for validation purposes, the EVs from *C. albicans* were isolated after the treatment with 1 mM caffeine, and their concentration was juxtaposed against a control group without any treatment (Figure 6). The comparison yielded no significant differences.

**Figure 7:**
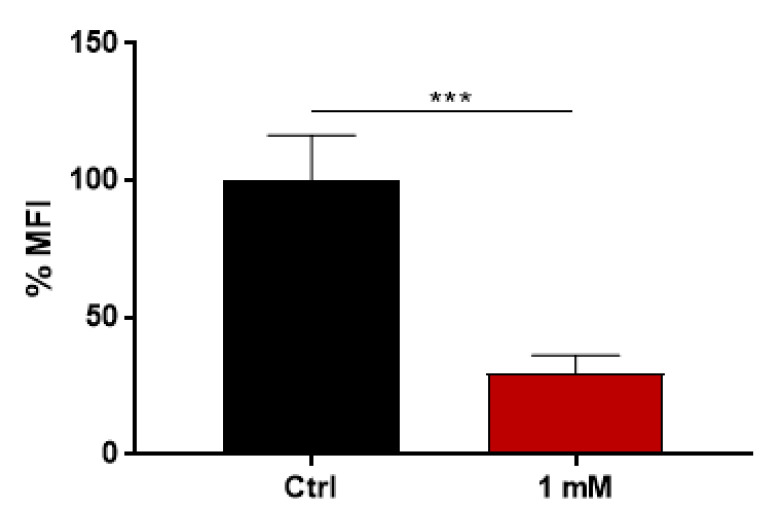
Chitinase activity of EVs isolated from caffeine-treated *H. capsulatum*. EVs isolated from 1 mM caffeine-treated *H. capsulatum* had significantly lower chitinolytic activity than those from the control group. Statistical analysis performed via Student’s *t*-test.; *** *p* < 0.001.

**Figure 8:**
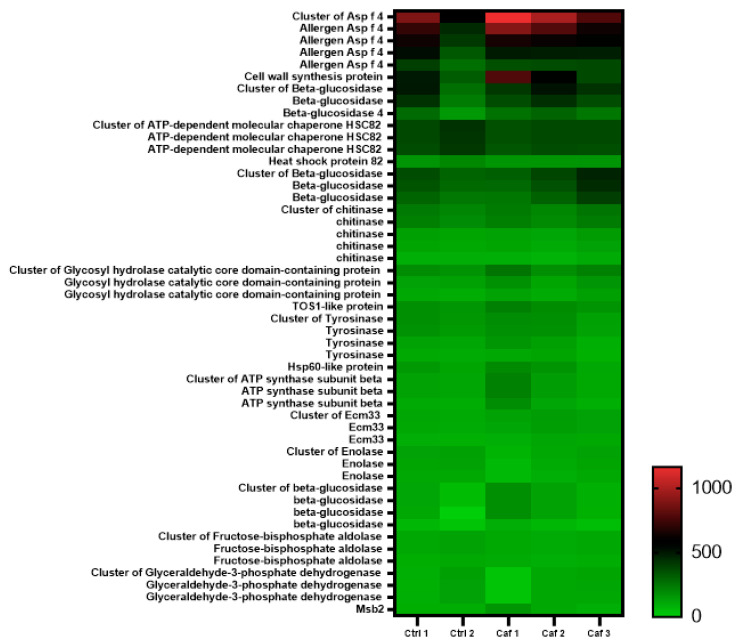
Proteomic profile unaffected by chitinase inhibition. EVs isolated from yeasts of the control group (absence of treatment—Ctr 1 and -2) and caffeine-treated group (1 mM caffeine—Caf1, -2 and -3) exhibited no significant differences in their overall proteomic profile. The displayed image highlights the 110 most prominently expressed proteins out of a total of 1249 identified proteins. Detailed data on these proteins can be accessed in Supplementary Table S1.

We also observed that vesicles isolated from the group treated with 1 mM caffeine also displayed decreased chitinolytic activity. This indicates that the treatment either affected the protein loading of the EVs or influenced the chitinase activity (Figure 7). Consequently, a proteomic analysis of EVs isolated from both the control and 1 mM caffeine-treated groups was performed to determine potential variations in EV cargo and identify any indications of cellular stress due to caffeine’s pleiotropic effects. Our findings reveal no significant proteomic differences between the EVs from both sample sets (Figure 8). Moreover, proteins linked to cell wall remodeling, such as chitinases and glucanases, were consistently expressed in both conditions (Table 1). Specifically, the chitinase CTS1 was comparably represented in both groups, highlighting that caffeine treatment did not alter its loading.

**Figure 9:**
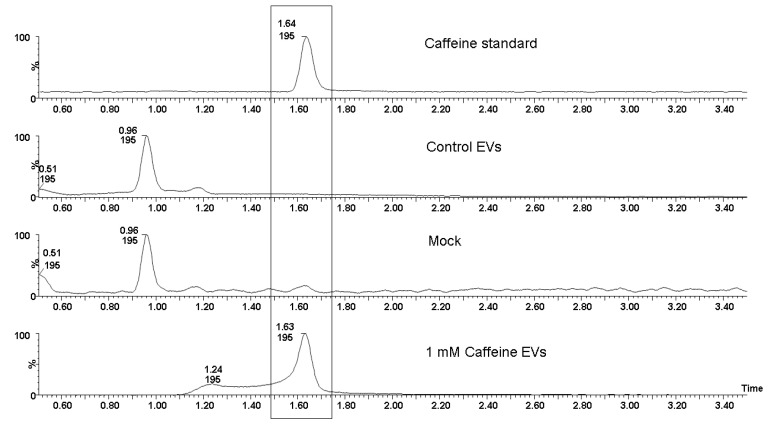
Presence of caffeine in *H. capsulatum* EVs. Qualitative HPLC-MS/MS analysis of the EVs isolated from both the control and the 1 mM caffeine-treated groups. When the retention times were compared with a known authentic caffeine standard, it was evident that only EVs from the caffeine-treated yeasts contained caffeine. In contrast, EVs from the control group showed no detectable caffeine.

Given the consistent chitinase levels in the EVs, HPLC-MS/MS analysis was employed to examine whether caffeine was transported within these vesicles after exposure, possibly accounting for the sustained inhibition observed. Our analysis confirmed the presence of caffeine in the EVs from the treated group (Figure 9).

### 3.5. H. capsulatum EVs Regulate Virulence in Galleria Mellonella

**Figure 10:**
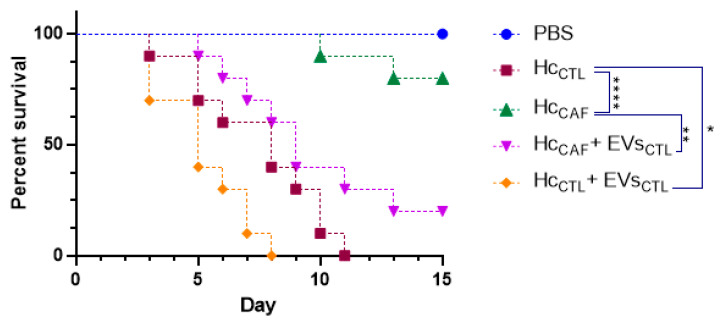
Impact of the treatment on *Galleria mellonella* survival rates. Infection of *G. mellonella* with a lethal inoculum of *H. capsulatum* caused death in 100% of the insects within 11 days (brown square). Conversely, infection with *H. capsulatum* yeasts treated with 1 mM caffeine reduced mortality by 80% (green triangle), with the first larvae dying only after 10 days of infection. *G. mellonella* co-infected with *H. capsulatum* and EVs isolated from the control group (orange diamond) showed a statistically significant increase in mortality. Mortality was restored when *G. mellonella* larvae were infected with *H. capsulatum* yeasts and EVs isolated from the control group (inverted purple triangle). PBS alone was used as a control with no effect on larvae (blue circle). A total of 10 larvae were used in each group. This figure is a representative experiment out of two independent experiments. Statistical analysis performed via log-rank Mantel–Cox; *, *p* < 0.05; **, *p* < 0.01; ****, *p* < 0.0001.

We employed the *G. mellonella* larvae as a model to gauge the effect of caffeine treatment on the virulence of *H. capsulatum*. Upon infecting the *G. mellonella* larvae with a lethal inoculum of *H. capsulatum* yeasts, there was a marked melanization, leading to 100% mortality within a span of 11 days (Figure 10). However, a stark contrast was noted when the larvae were infected with an inoculum of *H. capsulatum* yeasts previously cultivated in a Ham’s F-12 medium supplemented with 1 mM caffeine. The mortality rates plummeted to 20% over the experimental period, with the first larvae dying only after 10 days post-infection (Figure 10). To evaluate the direct influence of EVs released by *H. capsulatum* on the larvae, insects were co-injected with both a lethal inoculum of yeasts and EVs isolated from the control group (without caffeine treatment). Comparison mortality rates in these larvae versus those infected with only *H. capsulatum* revealed that the co-injection with EVs from the caffeine-free medium accelerated the death rate. Notably, when *G. mellonella* were co-infected with caffeine-treated *H. capsulatum* yeasts and EVs from the untreated group, the observed virulence mirrored that of the untreated *H. capsulatum* yeasts (Figure 10).

## 4. Discussion

EVs are ubiquitous molecular carriers present in nearly all cell types. These vesicles, enveloped by a lipid bilayer, house a diverse array of components including lipids, polysaccharides, glucans, nucleic acids, proteins, pigments and various metabolites [14,15]. In other organisms, EVs have been studied since the 60s, but fungal EVs were only first characterized in 2007 by our group and collaborators [8,13]. Many factors contributed to the delayed recognition of these vesicles, with the presence of the fungal cell wall making the existence of such compartments appear implausible before they were discovered. Over a decade since the initial discovery of fungal EVs, the processes by which they navigate through the cell wall remain enigmatic. Various theories have been proposed to explain this phenomenon. One prominent hypothesis posits that lytic enzymes contained within the EVs, specifically chitinase, might facilitate cell wall remodeling, thereby easing the transit of these vesicles through the fungal cell wall [8,45].

In an earlier study performed by our group, we demonstrated that the exposure pattern of chitin–oligomers in *H. capsulatum* yeasts was significantly enhanced compared to other yeasts of medical relevance [35]. In fact, fluorescence microscopy using rhodamine-labeled WGA (a lectin that binds specifically to *N*-acetyl-*D*-glucosamine oligomers) revealed that *H. capsulatum* displayed a homogeneous ring pattern of WGA, which co-localized with Uvitex 2B (a fluorescent probe that binds to chitin on the cell wall). In contrast, labeling in *C. albicans*, *C. parapsilosis* and *S. cerevisiae* resulted in punctuated WGA binding at the budding neck (Figure 1). These results suggested that the *H. capsulatum* increased exposure of chitin oligomers could be a consequence of an augmented chitinase activity in this yeast. Chitin, one of the major components of the fungal cell wall, would be broken down to chitin–oligomers, thereby enhancing its exposure. This hypothesis was further supported by a previous proteomic study conducted by our group, which revealed the presence of chitinase in *H. capsulatum* EVs [20]. The experiments conducted here not only corroborated the findings of the previous proteomic analysis, but also supplied direct evidence of chitinase activity in *H. capsulatum* EVs. 

Despite being highly abundant in fungi, the functional diversity of chitinases is not completely understood. Overall, their distinct biological roles include morphogenesis in yeast and mycelial forms, autolysis and chitin acquisition for nutritional purposes [28]. They are undoubtedly important to the cell wall remodeling process, being involved in several steps during different stages of the fungus life cycle, influencing the integrity of the cell wall, cell separation and cell wall resistance to stress in general [27]. There is a high degree of functional redundancy in fungal chitinases, hampering investigations about its individual functions and generating the need for several mutants with multiple-gene deletions to observe its respective phenotypical variations [27]. This, in addition to the fact that the positive and negative selections of *H. capsulatum* mutants require long periods, makes the use of pharmacological inhibitors for fungal enzymes such as chitinases attractive due to their rapidity, relative low cost, high specificity and the ability to be used for multiple strains [46,47].

Based on these findings, we conducted experiments using methylxanthines, a class of potent chitinase-competitive inhibitors [30], and found that aminophylline, caffeine and pentoxifylline were capable of inhibiting chitinolytic activity detected in *H. capsulatum* EVs at a 5 mM concentration. Caffeine and pentoxifylline presented optimal inhibition, while aminophylline presented only 70% of chitinolytic inhibition (Figure 2B). With optimal inhibition, caffeine was the chosen molecule for our subsequent experiments, showing that the inhibitory effect in *H. capsulatum* culture supernatant occurred in a dose-dependent fashion, up to 80% in the 5 mM concentration (Figure 2C), and that the inhibition was stable for at least 48 h (Figure 2D). The stability of inhibition over time was particularly important given that the standard protocol for EV isolation requires a 48 h culture period. It allowed us to isolate and compare EVs released under normal (control) conditions, with EVs produced when chitinase activity was inhibited. Likewise, although the antifungal activity of methylxanthines was previously observed at high concentrations [31], it was in our interest to determine a concentration that did not exhibit fungicidal or fungistatic effects. This was important because yeasts with impaired growth and viability would likely display a distinct EV release profile, distorting the comparison between the groups.

The growth curves (Figure 3) and SEM analysis (Figure 4) demonstrated that 1 mM caffeine had no effect on yeast proliferation nor differences in morphology when compared with the control group. Cells treated with 5 mM caffeine displayed defects in cell separation with the formation of multicellular aggregates (Figure 4), consistent with what was previously reported in studies involving the pharmacological chitinase inhibition in *S. cerevisiae* [48] and deletions of chitinase genes in *C. albicans* [49,50]. Notably, the literature reports a broad range of pleiotropic effects caused by caffeine in *S. cerevisiae*, some of which were related to cell wall stress pathways, but these effects were only present when high doses of caffeine (>10 mM) were used [51].

Our results showed that although treatment with caffeine did not affect the morphology or median size of the released EVs, EV release itself was dampened by 50% in the treated group (Figure 5). These results support our hypothesis that cell wall remodeling through EV enzymatic content is one of the mechanisms involved in their release. The fact that enzymatic inhibition only reduced EV release to 50% also suggests that this purported mechanism acts in tandem with other mechanisms previously hypothesized [8]. Alternatively, other non-excluding explanations would revolve around the participation of other hydrolytic enzymes, such as glucanases, in this process. To provide additional evidence that caffeine specifically inhibits chitinase activity under the conditions of our study, we examined its impact on the release of *C. albicans* EVs, which have a significantly lower chitinase content. NTA did not show differences in the amount of EVs released by both groups (Figure 6), suggesting either that chitinase might not be critical for EV release in *C. albicans* and/or that the “key enzyme” is species-specific, while also providing further evidence that the phenomenon observed in *H. capsulatum* is a direct result of chitinase inhibition and not a pleiotropic effect of caffeine.

The inhibition of EV release is particularly interesting because despite multiple biological effects being associated with different fungal EVs throughout the years [9,17,20], their biological roles still need to be elucidated. The broad range of biomolecules carried by EVs, many of those directly related to virulence, suggest that these compartments play a role in pathogenesis [15]. Additionally, the diminished release of EVs raised the question of whether their loading was altered, especially because the chitinolytic activity of the EVs isolated from the 1 mM caffeine group was also diminished (Figure 7), suggesting that protein loading into the EVs was somehow altered due to the pleiotropic effects of caffeine. Based on that, proteomic analysis was performed to investigate the possible differences in EV cargo in the control and 1 mM caffeine group. Our results demonstrate that the overall protein cargo of EV remains homogeneous between both groups (Figure 8), and the proteins associated with virulence, such as Hsp-60, and hydrolases associated with cell wall remodeling were equally expressed (Table 1). Among those, chitinase CTS1, for example, was also similarly expressed in EVs from both groups, providing evidence that the caffeine treatment did not have a marked impact on chitinase loading into the EVs, but successfully inhibited its chitinolytic activity. HPLC-MS/MS analysis (Figure 9) confirmed that EVs isolated from the 1 mM caffeine group carry the methylxanthine within their content, which explains the sustained inhibition of chitinolytic activity (Figure 7). It is worth noting that CTS1, from a total of eight different chitinases characterized in *H. capsulatum* [29], was the only chitinase found in its EVs, corroborating data from Baltazar et al. that identified CTS1 in *H. capsulatum* [21] EVs and Albuquerque et al. that found endochitinase [20]. CTS1 not only contains an extended serine/threonine-rich region common among extracellular proteins, but is also the only one of these chitinases that has a glycosylphosphatidylinositol (GPI) attachment site [29], consistent with what is described in the literature for proteins enriched in fungal EVs [52]. *H. capsulatum* CTS1 is also described in the literature to have a low, but constant, level of expression over several different growth conditions, consistent with it having an essential role in fungal growth and cell wall remodeling [29]. For the remaining seven chitinases described by Goughenour et al. not detected in *H. capsulatum* EVs, it is notable that CTS2 has the highest overall expression and is hypothesized to have a more general function. CTS3 is related to “self” chitinolytic activity related to hyphal growth, CTS4 is active, but less expressed and presumably acting under specific unknown conditions, CTS6 and -8 are hypothesized to be involved in the nutritional acquisition and degradation of environmental chitinase, and CTS5 and -7 have very low expression and little or no activity at all, suggesting that these two chitinases may no longer serve a functional role in *H. capsulatum* biology [29]. Taking this information together, it is no surprise that CTS1 was not only the only chitinase detected in EVs, but also seems to be responsible, at least partially, for their journey through the cell wall. 

It is also noteworthy that the dampened EV release observed upon chitinase inhibition can be related to both the inhibition of chitinases associated to the cell or EVs. CTS1 itself is also expected to be associated with the fungal cell wall. Since other chitinases could be involved, it is not possible, under our experimental conditions, to distinguish the nature of the inhibition observed. Nevertheless, both proteomic data (Figure 8 and Table 1) and EV release by *C. albicans* treated with caffeine (Figure 6) corroborate that the effect observed is directly associated with chitinase inhibition.

Since the production of EVs seems to be a constitutive process, the pharmacological inhibition of fungal EV release by caffeine provides a unique opportunity to gather direct insights into how these compartments are related to pathogenesis. To assess changes in virulence, we used the invertebrate infection model *G. mellonella*. Our results demonstrate that yeasts pre-treated with 1 mM caffeine lost their virulent profile, but co-inoculation with EVs was not only capable of recovering the virulence of those yeasts, but EVs effectively enhanced the virulence of non-treated yeasts (Figure 10), supporting the idea that *H. capsulatum* EVs indeed carry compounds that affect the fungal virulence. The ability of fungal EVs to facilitate pathogenesis in vivo has been described before in the literature, but only in a murine model in *C. neoformans* [24,26] and *S. brasiliensis* [53]. Although *G. mellonella* fungal infection has been extensively used to investigate the protective effects of EVs [9,10,14,17,25], it is important to highlight that the insect immune system is comparable only with the innate immune system of mammals [15,54,55], meaning that further investigation with more complex infection models is necessary. 

Taken together, our results demonstrated that chitinase inhibition by caffeine reduces EV release without altering their cargo or the fungus viability. Although there are a few molecules already described as effective EV release inhibitors in mammalian cells [56] and turbinmicin was shown to diminish EV release in *C. albicans* biofilms at concentrations that have antifungal activity [32], to our knowledge, this is the first account of a direct pharmacological inhibition to fungal EVs at concentrations below a direct antifungal effect. Furthermore, the dampened release of EVs has a direct effect on *H. capsulatum* virulence in vivo in the *G. mellonella* model, highlighting that, even though more research in the area is necessary, enzymes involved with fungal EV release can be explored as targets for novel antifungal therapies.

## Figures and Tables

**Table 1:** Consistent expression of hydrolases across samples. Various hydrolases, which play a role in the cell wall remodeling process, were detected and exhibited consistent expression levels in EVs from both the control and caffeine-treated groups across all replicates.

Identified Hydrolases	Accession Number	Alternate ID
Chitinase CTS1	A0A8H7Z0D3	CTS1
Beta-glucosidase I7I48_11962	A0A8H8CZ95	I7I48_11962
Beta-glucosidase I7I48_00930	A0A8H8D610	I7I48_00930
Beta-glucosidase I7I48_05879	A0A8H8D0Y1	I7I48_05879
Extracellular cell wall glucanase Crf1	A0A8H7Z116	I7I48_00580
Endoglucanase I7I48_00436	A0A8H8D5N0	I7I48_00436

## Data Availability

Not applicable.

## Supplementary Materials

The following supporting information can be downloaded at: https://www.mdpi.com/article/10.3390/jof9111052/s1, Table S1: Proteomics Raw Data.
